# The global carrier frequency and genetic prevalence of Upshaw-Schulman syndrome

**DOI:** 10.1186/s12863-021-01010-0

**Published:** 2021-11-17

**Authors:** Ting Zhao, Shanghua Fan, Liu Sun

**Affiliations:** 1 Department of Neurology, Henan Provincial People’s Hospital, People’s Hospital of Zhengzhou University, Zhengzhou, 450003 China; 2 Department of Neurology, Renmin Hospital of Wuhan University, Wuhan, 430060 China; 3 Yunnan Key Laboratory of Smart City and Cyberspace Security, Department of Information Technology, School of Mathematics and Information Technology, Yuxi Normal University, Yuxi, 653100 China

**Keywords:** Upshaw–Schulman syndrome (USS), Thrombotic thrombocytopenic purpura (TTP), ADAMTS13, Genetic prevalence, Pathogenicity, Carrier frequency

## Abstract

**Background:**

Upshaw–Schulman syndrome (USS) is an autosomal recessive disease characterized by thrombotic microangiopathies caused by pathogenic variants in ADAMTS13. We aimed to (1) curate the ADAMTS13 gene pathogenic variant dataset and (2) estimate the carrier frequency and genetic prevalence of USS using Genome Aggregation Database (gnomAD) data.

**Methods:**

Studies were comprehensively retrieved. All previously reported pathogenic ADAMTS13 variants were compiled and annotated with gnomAD allele frequencies. The pooled global and population-specific carrier frequencies and genetic prevalence of USS were calculated using the Hardy-Weinberg equation.

**Results:**

We mined reported disease-causing variants that were present in the gnomAD v2.1.1, filtered by allele frequency. The pathogenicity of variants was classified according to the American College of Medical Genetics and Genomics criteria. The genetic prevalence and carrier frequency of USS were 0.43 per 1 million (95% CI: [0.36, 0.55]) and 1.31 per 1 thousand population, respectively. When the novel pathogenic/likely pathogenic variants were included, the genetic prevalence and carrier frequency were 1.1 per 1 million (95% CI: [0.89, 1.37]) and 2.1 per 1 thousand population, respectively.

**Conclusions:**

The genetic prevalence and carrier frequency of USS were within the ranges of previous estimates.

**Supplementary Information:**

The online version contains supplementary material available at 10.1186/s12863-021-01010-0.

## Background

Upshaw–Schulman syndrome (USS) is an ultrarare but life-threatening autosomal recessive disease characterized by the absence or a severe deficiency of plasma von Willebrand factor (vWF)-cleaving protease; this results in the abnormal presence of ultralarge vWF multimers and subsequent platelet adhesion to these vWF multimers, leading to the formation of circulating platelet microthrombi [1–3]. The spectrum of clinical phenotypes in USS is broad. Disease onset can occur in the neonatal period, childhood, adulthood or late life, with a notable peak in women during pregnancy. Recurrent attacks of microvascular thrombosis with associated thrombocytopenia, purpura and microangiopathic haemolytic anaemia (MAHA) lead to ischaemic damage to end organs in the kidneys, heart, or brain. Diagnosis is based on a pentad of classic clinical characteristics: thrombocytopenia, haemolytic anaemia, renal failure, fever, and neurologic deficits [4, 5]. An ADAMTS13 activity assay combined with genetic testing distinguishes USS from acquired TTP. Treatment of USS involves the replacement of ADAMTS13 by fresh-frozen plasma (FFP) infusion.

USS is the result of homozygous or compound heterozygous variants in the ADAMTS13 gene. The ADAMTS13 gene spans 29 exons and ~ 37 kb, is located at chromosome 9q34 and encodes a protein with 1427 amino acids [6]. To date, more than 200 ADAMTS13 disease-causing mutations in all ADAMTS13 exons have been identified in patients with USS since 2001 [7–12].

USS is extremely rare, and its precise prevalence is uncertain. Most estimates suggest a prevalence of 0.5 to 2 cases per 1 million population. Previously reported prevalence rates of USS have been extremely heterogeneous; in central Norway, the prevalence was 16.7 per 1 million population, whereas in all of Norway, it was 3.1 per 1 million population, [13] which was 18 times and 3.4 times higher than the prevalence of USS in Japan (1 per 1.1 million population), [14] respectively. We hypothesized that the prevalence of USS would vary among different populations or ethnicities.

Therefore, we aimed to estimate the prevalence of USS across ethnicities from the current and largest publicly available Genome Aggregation Database (gnomAD) exome dataset using validated protocols [15, 16]. In addition, we aimed to generate an evidence-based dataset of known USS pathogenic variants via data mining. We also aimed to generate a machine learning training dataset for pathogenicity interpretation of variants.

## Methods

### Identification of known disease-causing variants

Literature was comprehensively reviewed to identify all known disease-causing variants in the ADAMTS13 gene (see the supplementary materials for search terms, protocols, scripts, full paper list and full variant list).

Two independent authors screened titles and abstracts according to inclusion and exclusion criteria: original case reports reporting disease-causing variants within the ADAMTS13 gene were included, and variants in full-text tables, figures or supplementary material figures and tables were extracted. Non-English-language articles, reviews, comments, editorials, etc.; nonoriginal papers; and in vitro and animal model studies were excluded.

All papers were saved in the Medline format and stored in the NoSQL database as MongoDB documents using NCBI Entrez Programming Utilities [17] (E-utilities) with the Python package biopython [18] and pymongo implementation.

The HGMD [19] (http://www.hgmd.cf.ac.uk/ac/index.php), Ensembl Variation [20], VarSome [21] (https://varsome.com/), ClinVar [22] (https://www.ncbi.nlm.nih.gov/clinvar/) and Genomenon Mastermind [23] (https://mastermind.genomenon.com/) databases were also searched to identify additional ADAMTS13 variants with reported pathogenicity.

A list of all single-nucleotide variants (SNVs) for ADAMTS13 was compiled using Ensembl Variant Simulator [24].

### Identification of major functional variants

The gnomAD [25] was searched for pathogenic variants that had not yet been reported in patients, and we examined major all-cause functional or structural changes (frameshifts, stop codons, start codons, splice donors and splice acceptors).

### Annotation of variants with allele frequency and functional predictions

Raw variants were identified and converted to Human Genome Variation Society (HGVS) nomenclature [26] using Mutalyzer [27] and Ensembl VEP Variant Recoder REST API with Python implementation. Ensembl variant effect predictor (VEP) [28] was used to annotate variants and make in silico predictions of pathogenicity with PROVEAN/PolyPhen/MutationTaster. gnomAD minor allele frequency (MAF) data were added to each variant from the gnomAD website.

### Disease-causing variant classification

The pathogenicity of variants was interpreted using a pipeline proposed by Zhang et al. [29] Disease-causing variants with gnomAD allele frequencies were classified using the American College of Medical Genetics and Genomics (ACMG) and the Association for Molecular Pathology (AMP) criteria [30] with the ClinGen Pathogenicity Calculator [31]. Pathogenic/likely pathogenic variants were included in the prevalence calculation.

### Maximum allele frequency filtering

All variants with gnomAD allele frequency data were filtered using a method defined by Whiffin et al. [32] Prevalence was calculated from estimates from the Japanese [14] population and Orphanet database as one case per 1 million population. The maximum allelic contribution was set at 24.4% based on an estimate of c.4143dup (p. Glu1382Argfs*6) according to International Hereditary Thrombotic Thrombocytopenic Purpura Registry [7] data. The maximum genetic contribution was set to 1 based on cohorts from the UK [8], France [9], and Germany [10] and International Hereditary Thrombotic Thrombocytopenic Purpura Registry [7] data. The penetrance was set at 50%, as suggested by Whiffin. The maximum credible allele frequency in the population was calculated as 0.035% by Whiffin’s defined equation.

The maximum allele frequencies for the population were directly downloaded from the gnomAD website (https://gnomad.broadinstitute.org/). Variants with a maximum allele frequency greater than the maximum credible allele frequency were excluded.

### Prevalence calculation

Allele frequencies of pathogenic/likely pathogenic variants were extracted from the ADAMTS13 variant dataset and pooled, and the prevalence of USS was calculated using the Hardy-Weinberg equation.

The 95% confidence interval (95% CI) for the binomial proportion was calculated using the Wilson score with the Python scientific computing package statsmodels and NumPy implementation. Graphics were plotted using the R packages ggplot2 and VennDiagram [33].

## Results

### Identification of *ADAMTS13* variants

**Fig. 1:**
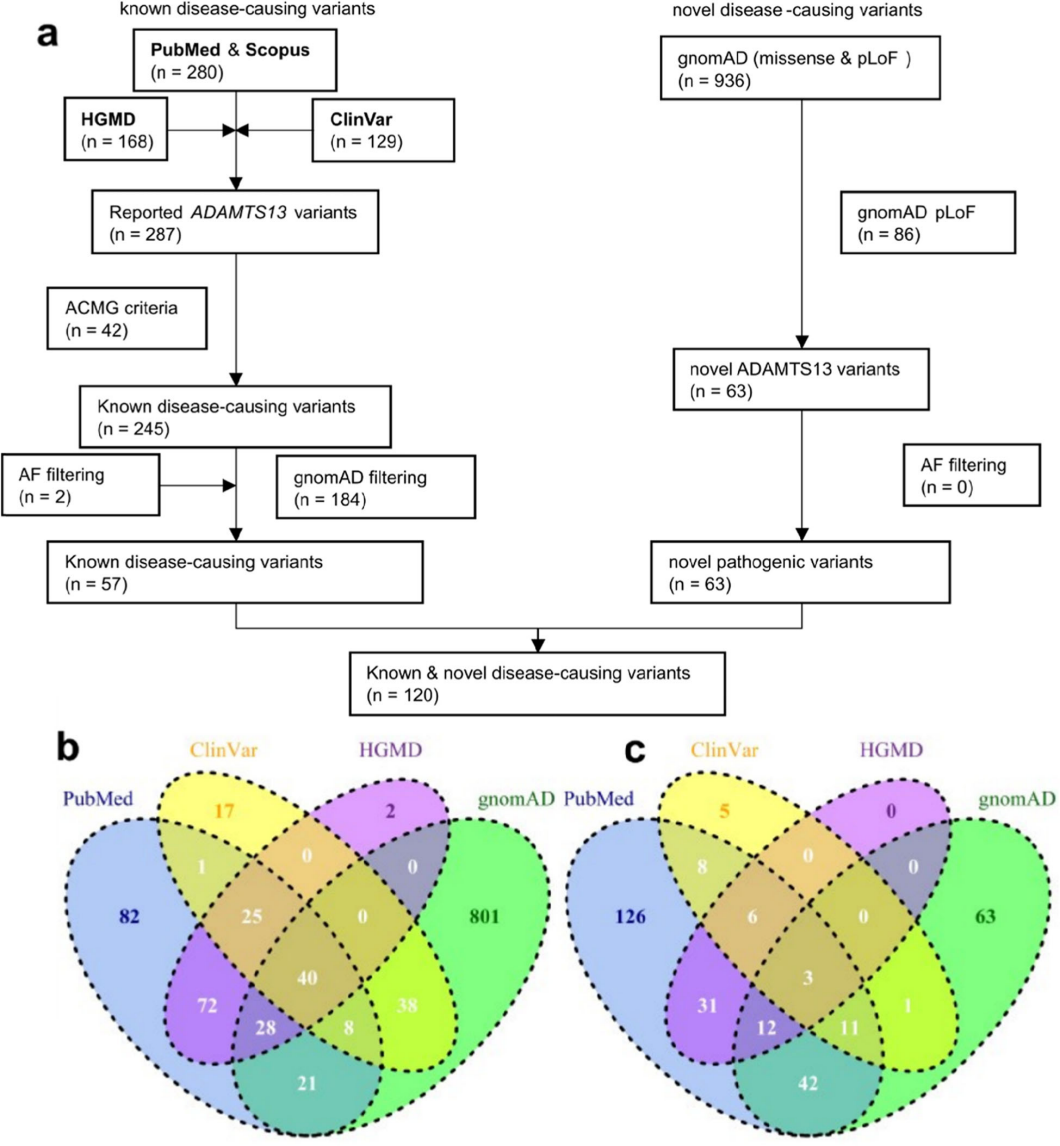
ADAMTS13 gene disease-causing variants and gnomAD allele frequencies. **a** flow chart of identification and classification of ADAMTS13 disease-causing variants. ADAMTS13 variants were extracted from PubMed & Scopus citations. ADAMTS13 missense, nonsense, frameshift, inframe, splice acceptor / donor variants were collected from HGMD Public (2016 version), ClinVar and gnomAD database. **b** Venn diagram of mined PubMed & Scopus, HGMD, ClinVar and gnomAD variants. **c** Venn diagram of mined PubMed & Scopus, HGMD, ClinVar and gnomAD disease-causing variants

Comprehensive searching for USS disease-causing variants resulted in the identification of 1249 articles, of which 126 studies were considered eligible according to the exclusion and inclusion criteria. From these studies, 280 disease-causing variants were identified, of which 239 variants were classified as “pathogenic” or “likely pathogenic” according to the ACMG criteria. Mining the ClinVar database resulted in the identification of an additional 6 disease-causing variants (pathogenic and likely pathogenic). A total of 245 known disease-causing variants were recorded. gnomAD allele frequencies were available for 59/245 (24.1%) disease-causing variants. All disease-causing variant pipelines and counts are shown in Fig. 1, and the associated data are shown in the supplementary data [see Additional files 1, 2, 3, 4, 5, 6, 7, 8, 9, and 10].

### Frequencies of reported USS pathogenic/likely pathogenic variants

**Fig. 2:**
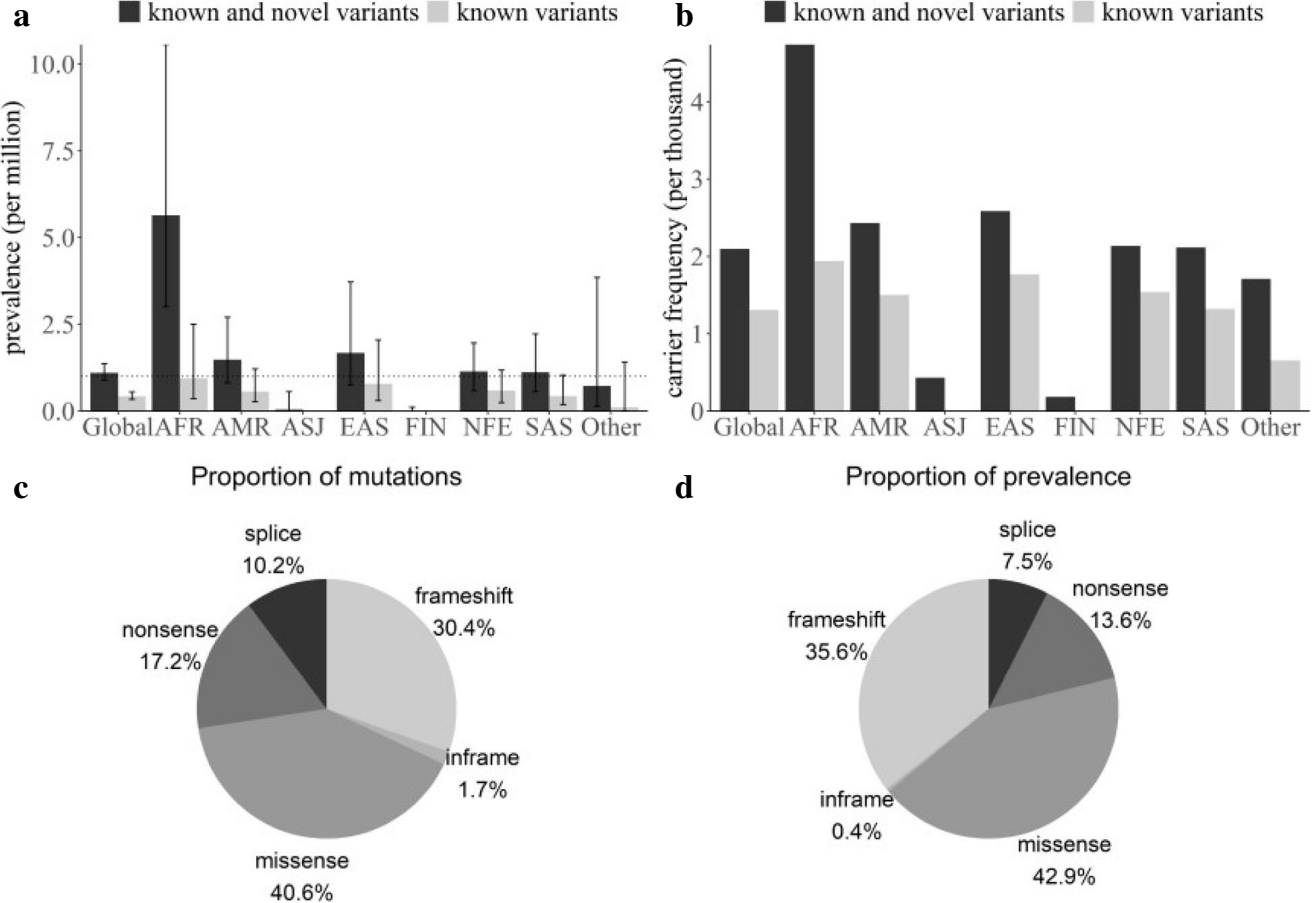
genetic prevalence and carrier frequency of USS. **a**, **b** USS carrier frequency and genetic prevalence estimated from gnomAD allele frequencies. **c**, **d** molecular consequence of all known and novel disease-causing variants. **c** Pie chart of the number of variants group by each molecular consequence. **d** Pie chart of the proportion of the total allele frequency group by molecular consequence

Of the 59 reported disease-causing variants with gnomAD allele frequency data, 57 remained after frequency filtering. Pooling of the allele frequencies of these variants resulted in a global allele frequency of 0.0006, which is equivalent to a prevalence of 0.43 per 1,000,000 population (95% confidence interval: [0.36, 0.55]). Five major populations had a similar prevalence of less than 1 per 1 million population (Fig. 2 and Table 1).

**Table 1:** Allele frequency database prevalence and carrier frequency calculations

	prevalence		carrier frequency	
	known and novel variants	known variants	known and novel variants	known variants
**total**	1.10152 (0.890567, 1.370326)	0.428407 (0.3357, 0.554897)	0.002097	0.001308
**AFR**	5.639105 (3.010004, 10.55961)	0.944298 (0.355737, 2.505441)	0.004738	0.001942
**AMR**	1.482111 (0.812177, 2.704048)	0.565474 (0.263468, 1.213389)	0.002432	0.001503
**ASJ**	0.046311 (0.003816, 0.561623)	0	0.00043	0
**EAS**	1.676507 (0.755126, 3.720573)	0.781969 (0.298701, 2.046257)	0.002586	0.001767
**FIN**	0.00864 (0.000654, 0.114046)	0	0.000186	0
**NFE**	1.143383 (0.595458, 1.961721)	0.593037 (0.239053, 1.177138)	0.002136	0.001539
**SAS**	1.121036 (0.56517, 2.22306)	0.436036 (0.183617, 1.035195)	0.002115	0.00132
**OTH**	0.731709 (0.138862, 3.850784)	0.107322 (0.008125, 1.415878)	0.001709	0.000655

*AFR* African/African American, *AMR* Latino/Mixed American, *ASJ* Ashkenazi Jewish, *EAS* East Asian, *FIN* Finnish, *NFE* Non-Finnish European, *SAS* South Asian, *OTH* Other

### Functional pathogenic variants

To estimate the genetic prevalence of USS, including disease-causing variants that had not yet been reported in patients, we searched all ADAMTS13 variants in the gnomAD database that caused loss-of-function (LoF) mutations (frameshift, nonsense, splice acceptor and splice donor variants). After filtering, 86 variants were identified in the gnomAD exome v2.1.1 database, and 63 variants were novel. When the novel disease-causing variants were combined with the reported pathogenic variants, and the global allele frequency of USS was 0.001, equivalent to 1.1 per 1,000,000 population (95% confidence interval: [0.89, 1.37]). The African population had the highest prevalence, at 5.64 per 1,000,000 population (95% CI: [3.01, 10.56]), and the other four major populations had a prevalence of greater than 1 per 1,000,000 population.

The most common functional mutation was a missense mutation, accounting for 40.6% of all pathogenic and likely pathogenic variants and contributing 42.9% of the total allele frequency. Frameshift and nonsense mutations were the second most common mutations.

## Discussion

We conducted the first systematic study to estimate, without bias, the genetic prevalence of USS in the global and five major populations. Our result was within the range of previous estimates. Additionally, we manually compiled all ADAMTS13 disease-causing variants and conducted an evidence-based interpretation of pathogenicity.

USS accounts for < 5% of TTP cases and is caused mostly by biallelic (compound heterozygote or homozygote) mutations in the ADAMTS13 gene or, in rare cases, by monoallelic ADAMTS13 mutations associated with single-nucleotide polymorphisms (SNPs). USS has a heterogeneous inheritance pattern. Previous estimates of USS prevalence were variable, which may be largely accounted for by differences in populations. Using the current largest population genome dataset in the gnomAD v2.1.1 (125,748 human exomes and 15,708 genomes), we calculated the global genetic prevalence of USS to be 0.43 to 1.1 per 1 million population and the carrier frequency to be 1 to 2 per 1 thousand population. We highlighted that the African population has the highest prevalence of USS, and the other four major populations have similar prevalence rates and carrier frequencies.

USS was not on the first Rare Diseases List released by the Chinese government [34]. The prevalence of USS in the Chinese population has not been estimated [35]. We have demonstrated the power and limitations of population genome datasets to calculate the genetic prevalence and carrier frequency of USS. The gnomAD groups East Asian populations into three categories: Korean, Japanese and other East Asians. Other population genome datasets, the 100 k Chinese People Genome Project and GenomeAsia 100 K Project will fill this gap [36]. We will estimate the prevalence of USS in Asian populations and Chinese populations with 100 k genome datasets as a next step.

Two variants, c.3178C > T (p. Arg1060Trp) and c.559G > C (p. Asp187His), which were classified as pathogenic and likely pathogenic, respectively, were filtered out by Whiffin’s method; they were “too common” to be causative factors for USS based on our set value for maximum allelic contribution and prevalence. Whiffin’s method was not optimal but more persuasive than an arbitrary MAF cut-off threshold of 0.05 (ACMG benign stand-alone criteria).

This study was based on assumptions of the Hardy-Weinberg equation. However, consanguine marriage is popular in specific subpopulations (such as some populations in Africa and South Asia). In these populations, the genetic prevalence might be higher than the calculated values. In addition, only one genetic prevalence calculation algorithm was used. Other algorithms, such as product-based algorithms for allele matrices and Bayesian-based algorithms, have been used to calculate autosomal recessive inherited retinal diseases [37] and limb-girdle muscular dystrophy [38], respectively.

The number of ADAMTS13 classified variants in the ClinVar database was far less than the number of reported variants obtained via document retrieval and data mining, but the pathogenicity prediction tool used the ClinVar dataset as the training set. The Clinical Genome (ClinGen) allele registry can be used for variant evaluation and assertion. The dbNSFP database, which provides comprehensive functional prediction and annotation for human nonsynonymous and splice-site SNVs, is a valuable resource for training set construction for pathogenicity prediction of novel variants [39].

Our finding of reported disease-causing variants and predicted pathogenic variants highlight the mutational spectrum of USS. The most common pathogenic variants were missense variants, which were also the most difficult to predict and evaluate for pathogenicity. The data from this study can be used for the creation of toolboxes for geneticists, clinicians, genetic counsellors, and health data analysts.

In summary, the genetic prevalence of USS was 0.43 per 1 million population (95% CI: [0.36, 0.55]) for the 239 known pathogenic/likely pathogenic variants and 1.1 per 1 million population (95% CI: [0.89, 1.37]) for the 245 (239 known and 6 novel) pathogenic/likely variants, which was calculated from the gnomAD containing 125,748 individuals with whole-exome sequence data and 15,708 individuals with whole-genome sequence data. These results are within the range of previous estimates a prevalence of 0.5 to 2 cases per million population from Kremer Hovinga JA et al. but different from those of other previous studies. The prevalence of USS in central Norway was 16.7 per 1 million population based on 11 cases of USS in central Norway, which has a population of 659,621 persons, and 3.1 per 1 million population based on 16 cases in all of Norway, which has a population of 5.17 million. However, Kokame et al. estimated a 6/3200 heterozygosity rate on the basis of 6 of 3200 samples, and the prevalence was 1 per 1.1 million population (6/3200 × 6/3200 × 1/4) in Japan, which was the same as that estimated from the Orphanet database. Furthermore, they estimated 110 USS patients in Japan based on a 0.13 billion population. The Norway study calculated the prevalence based on two variant allele frequencies, namely, c.4143dup and c.3178 C > T (p. R1060W), and the Japan study based the prevalence on seven variants. The estimation of the USS prevalence may be biased due to insufficient sample sizes, different ethnicities, different lethality, different penetrance, misdiagnosis, etc. We calculated more reliable global and population-specific estimates for USS genetic prevalence and carrier frequency. These data can be used as a training set for pathogenicity prediction of novel variants and genetic diagnosis of USS. We also provided a validated pipeline to calculate the prevalence of rare diseases. These datasets will be especially valuable for rare disease definitions in developing countries, in which epidemiological data are scarce [40].

## Supplementary Information

**Additional file 1.**

**Additional file 2: Supplemental Table S1.** All ADAMTS13 variants mined from literature.

**Additional file 3: Supplemental Table S2.** All ADAMTS13 reported variants.

**Additional file 4: Supplemental Table S3.** all database and reported variants collection from ClinVar, HGMD with gnomAD allele frequency.

**Additional file 5: Supplemental Table S4.** All ADAMTS13 variants collection.

**Additional file 6: Supplemental Table S5.** All ADAMTS13 variants collection with gnomAD allele frequency.

**Additional file 7: Supplemental Table S6.** Eight population ADAMTS13 genetic prevalence and carrier frequency.

**Additional file 8: Supplemental Table S7.** ADAMTS13 variants in ClinVar database.

**Additional file 9: Supplemental Table S8.** ADAMTS13 variants in gnomAD database.

**Additional file 10: Supplemental Table S9.** ADAMTS13 variants in HGMD database.

## Data Availability

The datasets are available in the Science Data Bank (ScienceDB) repository. 10.11922/sciencedb.00628

## Abbreviations

MAF: Minor allele frequency
ClinGen: Clinical Genome
ACMG: American College of Medical Genetics
USS: Upshaw-Schulman syndrome
TTP: Thrombotic thrombocytopenic purpura
